# Characterization of a Trpc6 Transgenic Mouse Associated with Early Onset FSGS

**DOI:** 10.9734/bjmmr/2015/12493

**Published:** 2014-10-30

**Authors:** Cesar P. Canales, Paola Krall, Pamela Kairath, Irene C. Perez, Miryam A. Fragoso, Paulina Carmona-Mora, Phillip Ruiz, Jochen Reiser, Juan I. Young, Katherina Walz

**Affiliations:** 1 John P. Hussman Institute for Human Genomics, University of Miami Leonard Miller School of Medicine, Miami, Florida, USA.; 2 Cellular and Genetic Medicine Unit, School of Medical Sciences, UNSW, Sydney, Australia.; 3 Nephrology Unit, Faculty of Medicine, Universidad Austral de Chile, Valdivia, Chile.; 4 School of Biochemistry, Faculty of Sciences, Universidad Austral de Chile, Valdivia, Chile.; 5 Department of Surgery and Pathology, University of Miami Leonard Miller School of Medicine, Miami, Florida, USA.; 6 Division of Nephrology and Hypertension, University of Miami Leonard Miller School of Medicine, Miami, Florida, USA.; 7 Department of Internal Medicine, Rush University, Chicago, USA.; 8 Department of Human Genetics, University of Miami Leonard Miller School of Medicine, Miami, Florida, USA.

**Keywords:** FSGS, TRPC6, glomerulopathies, podocytes, kidney disease, mouse models, transgenic, genetic bases of human diseases

## Abstract

**Rationale:**

Mutations in Transient Receptor Potential Channel 6 (*TRPC6*) gene are associated with autosomal dominant focal and segmental glomerulosclerosis (FSGS). The majority of the identified mutations affect the ion channel function. Since calcium channels are promising candidate drug targets, there is an an urgent need for a mouse model to assess new therapeutic drugs and to help delineate the pathogenic process leading to FSGS. We have previously reported the generation of three independent transgenic mouse lines carrying different Trpc6 mutations that display a glomerular disease comparable to the phenotype presented by individuals with FSGS. However, the utility of these models for drug testing is dampened by the late-onset of the presentation and the mild phenotypic manifestations.

**Methodology:**

In order to obtain a time-effective mouse model for Trpc6-associated FSGS we generated a new transgenic mutant Trpc6 mouse model emulating the amino acid change carried by the first pediatric patient of FSGS associated with a TRPC6 mutation: M132T.

**Results:**

Mice carrying the orthologous Trpc6 M131T transgene showed early onset proteinuria and early signs of FSGS. When exploring molecular consequences of the overexpression of this mutated form of Trpc6 in podocytes, differences in expression levels of Axin2 and β-catenin were found in glomeruli from transgenic Trpc6 M131T mice. These data supports the proposed molecular mechanisms related to the activation of calcineurin-NFAT/Wnt signaling, as outcome of the increased calcium influx caused by the mutated form of Trpc6.

**Conclusion:**

Given that the Trpc6 M131T mouse develops an early onset of FSGS-like phenotypes it represents a promising model for studying the pathogenesis of FSGS caused by TRpC6, facilitating the assessment of new drugs as treatments and allowing further studies to understand underlying molecular pathways involved in the development of the TRPC6 mediated disease.

## 1. INTRODUCTION

Focal and Segmental Glomerulosclerosis (FSGS) is a histological lesion, characterized by sclerosis in some glomeruli (focal) with partial (segmental) distribution. It is a common cause of chronic kidney disease and end-stage renal disease of rising incidence both in children and adults [1]. The hereditary form of FSGS has been reported to be caused by mutations in genes encoding different proteins expressed in podocytes: *NPHS1, NPHS2, WT1, ACTN4, CD2AP, PLCE1, LAMB, TRPC6, MYH9, INF2, MYOE1* and *PODXL* [2-8]. Podocytes are highly specialized cells and their structural integrity have to remain intact to properly function as part of the glomerular filtration barrier [9,10].

TRPC6 is a six transmembrane domain protein, member of the large transient receptor potential (TRP) superfamily of nonselective cation channels [11,12]. Although TRPC6 is ubiquitously expressed, its role in podocytes has become particularly interesting, since the identification of the first mutations associated to FSGS [5,13]. TRPC6 has been reported to be localized in the slit diaphragm, interacting with NPHS1, NPHS2 and CD2AP [13,14].

Up to date, an increasing number of mutations in Trpc6 have been described in familial and sporadic cases of FSGS [5,13,15-18]. However, this calcium channel seems to be involved not only in the genetic form of FSGS, but in other acquired glomerular diseases such as minimal change disease and membranous glomerulonephritis [19]. The calcium channel levels of the non-mutant form are also significantly up regulated in these diseases, and thus suggest the involvement of TRPC6 in the pathology of non-genetic forms of proteinuric disease [19,20]. Interestingly, mice transiently overexpressing Trpc6 showed an important increase of proteinuria, which is an early sign of glomerular disease associated with podocyte injury [19]. Consistently with these findings, we have previously reported that high levels of wild-type Trpc6 expressed specifically in podocytes also trigger a glomerular disease presenting with signs of FSGS [21]. Furthermore, podocyte-associated markers including TRPC6 have been found increased in diabetic nephropathy urine samples and correlated with albuminuria, suggesting that TRPC6 mRNA may represent another clinical biomarker of podocyturia in diabetic nephropathy [22].

Despite this strong evidence relating TRPC6 with different forms of kidney diseases, and the recent availability of specific drugs to modulate the TRPC6 activity [23], there is a lack of targeted treatments for Trpc6 related kidney diseases. Part of the problem is the lack of adequate animal models i.e. the late onset and the milder phenotype observed in the previously generated mouse models [21]. In order to address this difficulty, here we present the generation and characterization of a new mouse model for FSGS due to the Trpc6- M131T point mutation equivalent to the TRPC6-M132T mutation in humans that is associated with an early onset of the disease [15]. Consistently, our results for this model showed an early onset of the FSGS-related phenotype, in a similar mouse-timeframe proportion to what has been described in the human patient carrying the same aminoacidic change [15]. In addition, these mice presented a relatively stronger severity of the pathological signs found when compared to the models previously described by our group [21]. We propose the present Trpc6-M131T mouse model as a new tool to study the pathogenesis of FSGS caused by Trpc6, to assess the outcomes of drugs treatments and to facilitate studies of Trpc6-related molecular pathways.

## 2. MATERIALS AND METHODS

### 2.1 Cloning and Generation of Trpc6 M131T Mutant form

Point mutation M131T was generated upon the Trpc6-HA construct previously described [21]. Site directed mutagenesis from this construct containing the full length Trpc6 cDNA was performed using the following primers: Forward 5’- GTG GAT TAC ACC GGC CAG AAT G-3’ and Reverse 5’- CAT TCT GGC CGG TGT AAT CCA C −3’. The underlined nucleotides produce an aminoacidic change of Methionine by Threonine in position 131 (M132T in human). PCR products were cloned in pGEMT-Easy and then subcloned to pCDNA3 Trpc6-HA. The nucleotidic change was confirmed by direct sequencing.

### 2.2 *In vitro* Studies

To determine the ability of pCDNA3 *Trpc6-HA*M131T to express the proteins we performed Western blot and immunofluorescence analysis as described before [21]. Briefly, for Western blot HEK293 cells were transfected with the plasmid containing the Trpc6-HA M131T cDNA and lysed 16 hours after transfection. Expression was confirmed with rat anti-HA antibody 1/5000 (clone 3F10, Roche). To determine subcellular localization, HeLa cells were co-transfected with the same construct and method. Plasmid pDsRed Monomer-F (Clontech) was also transfected for membrane co-localization analysis. The same rat anti-HA antibody(1/500) was used for detecting Trpc6 M131T membrane signal. Mounted slides were analyzed with confocal Zeiss LSM710 microscope and its respective software.

### 2.3 Generation and Molecular Characterization of Transgenic Mice

As in our previous report the CMV promoter was replaced with the human podocin- promoter (NPHS2). To confirm the accuracy of the transgenes the plasmids were sequenced before microinjection. Transgene preparation, isolation and microinjection into pronuclei of C57B6/6J X CBA/J zygotes were performedin the mouse facility of the Centro de Estudios Científicos-CECsas described before [21]. Initially, three founders were selected for colony expansion by crossing them with pure C57B6/6J mice to obtain F1 mice. F1 x F1 mating were set up to generate F2 mice that were used for all the phenotypic characterization. Mice were maintained in a SPF facility with a 12 h light: dark cycle (lights on at 6 AM, off at 6 PM) with access to food and water *ad lib*. All animal work was approved by the Institutional Animal Care and Use Committee of the University of Miami. pNPHS2 Trpc6-HA wt F419 transgenic mice, also utilized in this work as transgenic over expressing the wild type Trpc6 specifically in podocytes, are available from The Jackson Laboratory as JAX Stock No. 018293.

Genotyping was performed from tail digested genomic DNA from 14-21 day old mice by PCR as described previously [21]. For dot blot analysis 10 μg of tail DNA were loaded into a Hybond C+ membrane and incubated with a probe that hybridizes to exon 2 of *Trpc6*. The specificity of the probe was previously assayed in a Southern blot analysis performed in our previous report [21].

### 2.4 Determination of Transcripts Expression

Pure glomeruli extracts were obtained with Dynabeads (Invitrogen) recovery from kidney after transcardial perfusion as previously described [24]. Total RNA was isolated from these fractions with TRIzol Reagent (Invitrogen) according to manufacturer’s instructions. Prior to reverse transcription, RNA samples were treated with rDNAse I (DNA-free kit, Ambion). Then cDNA was synthetized using ImProm-II Reverse Transcription System (Promega). The possibility of contamination with genomic DNA from the transgene was eliminated by using rDNAseI and ‘no RT’ controls in the reactions of Real Time PCR. Real time PCR reactions were performed with the Agilent Brilliant III Ultra-Fast SYBR Green QPCR Master Mix (Catalogue nr. 600882). Primers used for detecting *Trpc6* transcript were designed in exon 2 and normalized against *Gapdh* [21]. For quantification of *Axin2* mRNA levels the following primers were utilized Forward: 5’-CGA AGC ACG TTC ACC ACC ACT ACA T-3’ and Reverse: 5’-CCG ACA GTG CAA GAC CCG GT-3’. All reactions were performed in triplicate as follows: 3 min at 95°C, and 40 cycles of 5 s at 95°C and 10 sec at 58°C. *Gapdh* was utilized as a normalization control. ΔΔ Ct method was used to compare the ΔCt value of transgenic animal samples (Ct of target-Ct of control transcript) with ΔCt value of wild type mice samples.

### 2.5 *In vivo* Protein Expression Analysis

To confirm that the expression of the transgene was restricted to podocytes, an immunofluorescence analysis against HA epitope and Synaptopodin as a podocytes marker was performed. Adult mice were perfused with 1x PBS and 4% PFA. Kidneys were dissected and then frozen in OCT medium. Five μm sections were washed three times (5 minutes each) with 1x PBS and incubated for one hour at room temperature with blocking solution (1x PBS, 10% NGS, 0.3% Triton x-100). Additional blocking steps were performed using Super Block and Mouse-to-Mouse Blocking Reagent according to the manufacturer instructions (ScyTek Laboratories). Primary antibodies (prediluted in blocking solution) were incubated ON at 4 °C (Rabbit anti HA 1:300, Bethyl Cat. No. A190-108A; Mouse anti Synaptopodin 1:80, Progen Cat.No. 61094). Secondary antibodies were incubated for 40 minutes at RT (Goat anti Rabbit AF568 1:1000; Goat anti Mouse AF488 1:1000). Finally sections were washed with 1x PBS, rinsed with water and mounted with Dako Fluorescent Mounting Medium (Dako). Mounted slides were analyzed with Nikon Eclipse TE2000-U microscope. Pictures were captured with Q Imaging Fast 1394 digital camera using QCapture Pro software (Version 6.0.0.412). Merges were digitally processed using Adobe Photoshop 11.0.

Glomeruli extracts were prepared by isolating glomeruli through a sieving technique and homogenized in a tight potter with 30 strokes. Cellular fractions were prepared using the NEPER Nuclear and Cytoplasmic Extraction Reagent (Pierce) according to the manufacturer’s instructions. 10 micrograms of total protein of both the cytoplasmic and nuclear fractions were resolved on 12% SDS-PAGE gels and proteins were then transferred onto polyvinylidene fluoride (PVDF). Blots were probed with mouse anti β-catenin (1:1000, BD Transduction Laboratories), rabbit anti-acetyl H3 (1:7000, company) and rabbit anti-Gapdh (1:1000, Cell Signaling) was utilized as a normalization control. Incubation with horse radish peroxidase (HRP)-conjugated secondary antibodies was utilized for detection.

### 2.6 Phenotypic Characterization

#### 2.6.1 Proteinuria

In order to determinate the presence of an early phenotype minimizing the error due to sex or age differences all the phenotypic characterization was performed in young male mice at 2 months of age. Albuminuria was measured in fresh urine samples. Albuminuria normalized by creatininuria (μg/mg) was determined as described previously [21,25]. The number of mice analyzed for each genotype was as follows: non transgenic mice: n=9, Trpc6 WT over expressor n=11, Trpc6 E896K n=5, Trpc6 M131T n=14. The previously generated Trpc6 P111Q transgenic line was not included in this study due to transgene silencing.

#### 2.6.2 Histopathological analysis

Mice were transcardially perfused with 15ml of room temperature 1x PBS and then 30 ml of cold 4% PFA. Perfused kidney were dissected, dehydrated in alcohol gradient and embedded in paraffin. Four μm thick sections were used for periodic acid-Schiff reagent (PAS) staining. The number of mice analyzed for each genotype was as follows: non transgenic mice: n=9, Trpc6 over express or n=11, Trpc6 E896K n=5, Trpc6 M131T n=11. All the samples were examined by a pathologist in blind. The pathological abnormalities in the kidney were gradedbased on the severity of component abnormalities in glomeruli such as glomerulosclerosis, hypercellularity, and segmental mesangial expansion. The severity of each abnormality was scored based on a method previously described [26] where 0 represented absence of abnormalityand scores between 1 and 4 represented mild, moderate, moderately severe and severe abnormalities. Briefly, the scores for abnormality intensity (0-4) and abnormality extension (0-4) were multiplied to get final values that were analyzed by statistical test.

### 2.7 Statistical Analysis

Albuminuria/creatininuria values were normalized by log_10_ to perform ANOVA. Post hoc Tukey test was performed to determine pairwise statistical differences between each group of mice (non-transgenic wild-type, Trpc6 wild-type, Trpc6 E896K and Trpc6 M131T). Histopathology injury scores did not fit a normal distribution even after transformation. Then, Kruskal-Wallis tests were performed to detect differences between mice groups. Post hoc Wilcox test was performed to determine pairwise comparisons for non-normal data. Error bars represent standard errors of the mean (SEM). Values of *p*< 0.05 were considered to be significant.

## 3. RESULTS

### 3.1 Generation of Podocyte Specific Trpc6-HA M131T Transgenic Mice

**Fig. 1.**
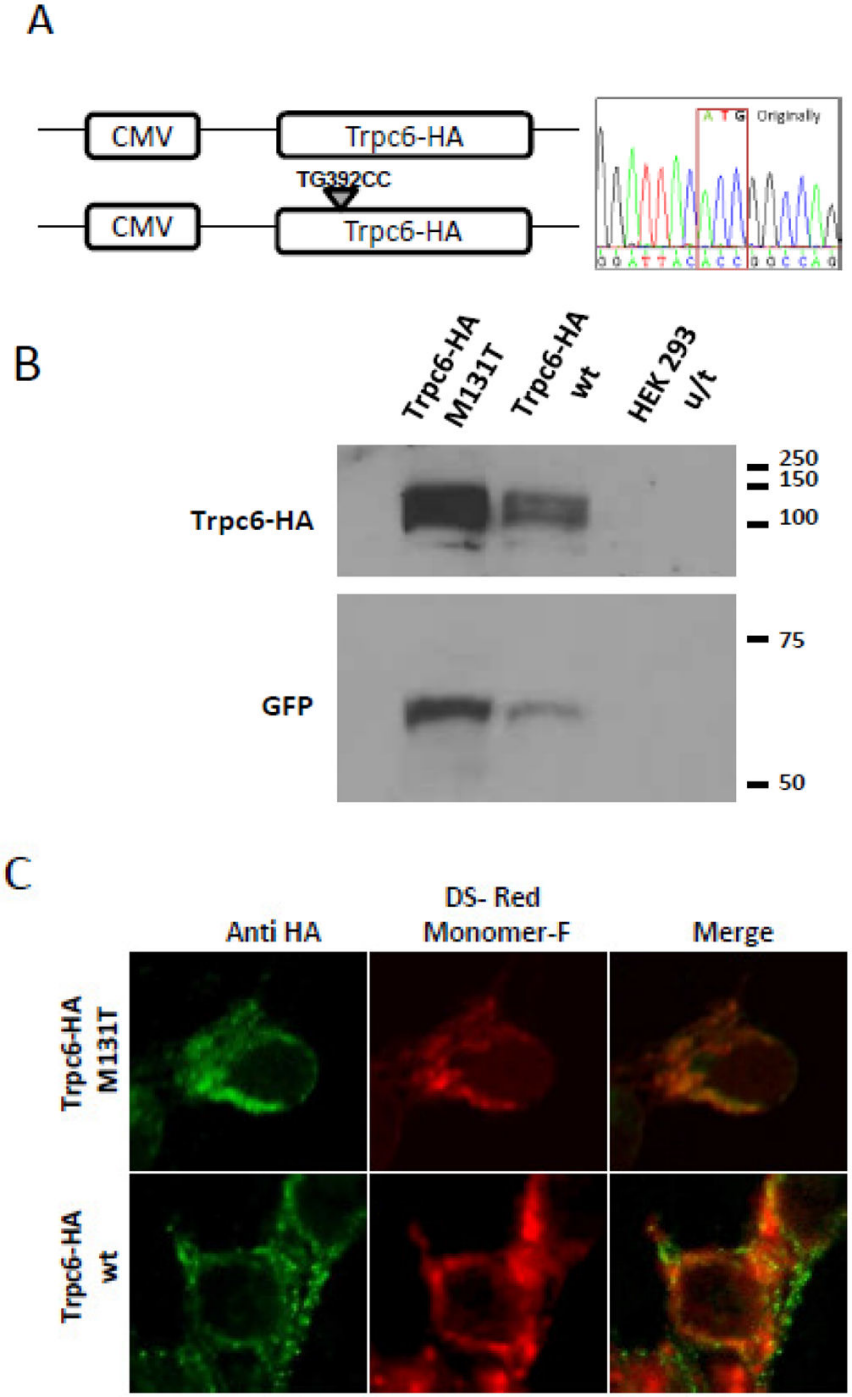
Generation and *in vitro* analysis of mutated Trpc6 M131T (A) Schematic representation of the Trpc6 constructs utilized for in vitro studies. The chromatogram pointing the nucleotide change that produces the M131 Tmutation is depicted. (B) Lysates from either unstransfected (u/t) EBNA293 cells or transfected with a plasmid containing Trpc6-HA M131T, Trpc6-HA wt were analyzed by Western Blot analysis with an antibody detecting either the tagged HA epitope (top), and GFP tag as a transfection control (bottom). (C) Subcellular localization of wild type and mutated forms of Trpc6 and plasma membrane. HeLa cells were co-transfected with pDsRed Monomer-F (plasma membrane marker) and a plasmid containing either Trpc6-HA M131T or Trpc6-HA wild type. Trpc6 (HA epitope in green) co-localizes with the plasma membrane marker (red) for all the transfected proteins. Images were obtained from a confocal microscope (630x)

A vector containing the full length mousewild-type *Trpc6* cDNA, tagged with a HA epitope at the C-terminal and expressing the calcium channel under the control of the CMV promoter was previously generated to test protein stability and subcellular localization [21]. In this study, we used site-directed mutagenesis to introduce the specific nucleotide change c.392_393TG>CC on the wild-type *Trpc6* cDNA, that would generate a mutant Trpc6 M131T protein (Fig. 1A). EBNA293 cells transiently transfected with the wild-type and mutant *Trpc6-HAM131T* cDNA confirm that the mutant protein is stable and displays the expected molecular weight. The molecular weight of both proteins (~106 KDa) was corroborated by Western Blot analysis using an antibody against HA that detected proteins of the expected molecular weight for Trpc6 in cell extracts from cells transfected with both constructs (Fig. 1B) indicating that the mutant protein has the correct molecular weight. *GFP* cDNA was expressed from the same plasmid, but under the control of an independent promoter, hence GFP was utilized as transfection control (Fig. 1B). No differences were observed in the ratio of expression for both the wild type and the mutant protein vs GFP, suggesting the same levels of stability for the wild type and the mutant protein. The subcellular localization of the modified protein was also assayed by co-transfecting Trpc6-HA and the plasma membrane marker pDsRed Monomer-F into HeLa cells. Trpc6-HA M131T co-localizes with the membrane protein, indicative of correct and expected subcellular localization in the same way as the wild type Trpc6 (Fig. 1C).We have previously showed that the addition of the HA tag did not affect the functionality of the Trpc6 channel [21].

**Fig. 2.**
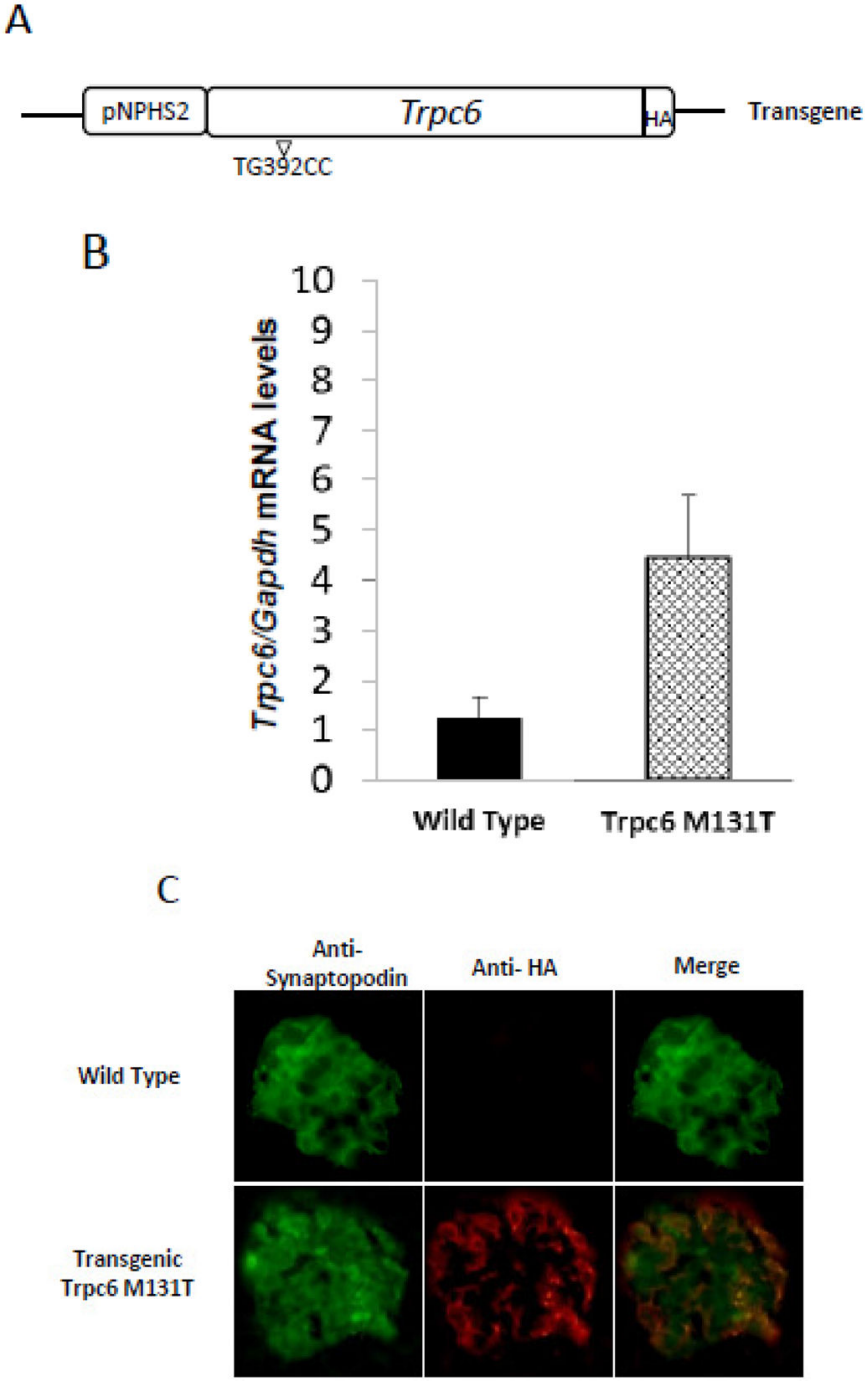
Molecular characterization of the *Trpc6-M131T* transgenic line (A)Scheme of the microinjected transgene Trpc6- M131T and its comparison with the wild type allele. The complete Trpc6-M131T cDNA were subcloned downstream the pNPHS2 podocin promoter as previously described [21]. (B) Relative mRNA Trpc6/Gapdh expression levels in glomeruli were determined by real-time PCR. Values represent mean +/− SEM; n=5. (C) Immunofluorescence in kidney cryosections showing podocyte-specific Trpc6-M131T transgen expression. A podocyte specific marker, Synaptopodin (green) was used as control to determine HA (red) HA (400x)

In order to achieve podocyte-specific transgene expression, the cDNA of Trpc6-HAM131T was subcloned down stream of the podocin (pNPHS2) specific promoter (Fig. 2A). By pronuclear microinjection, 5 transgenic mice were obtained for Trpc6-HA M131T. Two founders were randomly selected and crossed with C57BL/6J wild-type mice (designated as lines 367 and 395 according to the founder’s identification). The copy number of the inserted transgene was estimated by Dot Blot analysis using a probe directed to exon 2 of mouse Trpc6 that has shown to produce a specific signal for the transgene ([21], data not shown). The determined copy numbers were 4 and 12 for lines 367 and 395, respectively [21]. The Trpc6 mRNA expression levels for each line were estimated at the age of two months by quantitative real-time PCR from an enriched glomeruli fraction obtained with the Dynabeads and sieving technique [24]. The line 367 with an mRNA expression level of 4.2*+/−* 0.5 times more than the endogenous *Trpc6* mRNA, measured in wild type mice, was selected for further studies (Fig. 2B). To confirm the protein expression in podocytes we performed indirect immunofluorescence of frozen kidney sections utilizing an antibody against Synaptopodin (a podocyte-specific marker) [27], and an anti-HA antibody to detect the transgenic tagged protein. Expression of the transgene was observed in the glomerular podocytes, as illustrated by a consistent co-localization with Synaptopodin in glomeruli of transgenic Trpc6 M131T mice (Fig. 2C).

### 3.2 Phenotypic Characterization of Trpc6 M131T Transgenic Mice Shows a More Severe Pathological Phenotype When Compared to Other Trpc6 Transgenic Lines

In order to be able to characterize the phenotype of Trpc6 M131T transgenic miceand to compare it with other kidney mutant lines of Trpc6, we have included in the phenotypic analysis two different transgenic lines: one that over expresses the wild type form of Trpc6 (named as Trpc6 WT) and another one that overexpresses the mutant Trpc6 E986K (named as Trpc6 E896K). In all cases the over expression of Trpc6 is directed by the pNPHS2 promoter, hence restricted to podocytes. For these lines the transgene copy number was: 2 for Trpc6 WT and 17 for Trpc6 E896K. The levels of expression of the transgene are: 2.0*+/−*0.9 for Trpc6 WT and 2.5*+/−*0.1 for the Trpc6 E896K mice [21]. Despite the difference in transgene copy numbers, the levels of expression of the transgenes are similar among the three transgenic mouse lines allowing the phenotypic comparison between them.

**Fig. 3.**
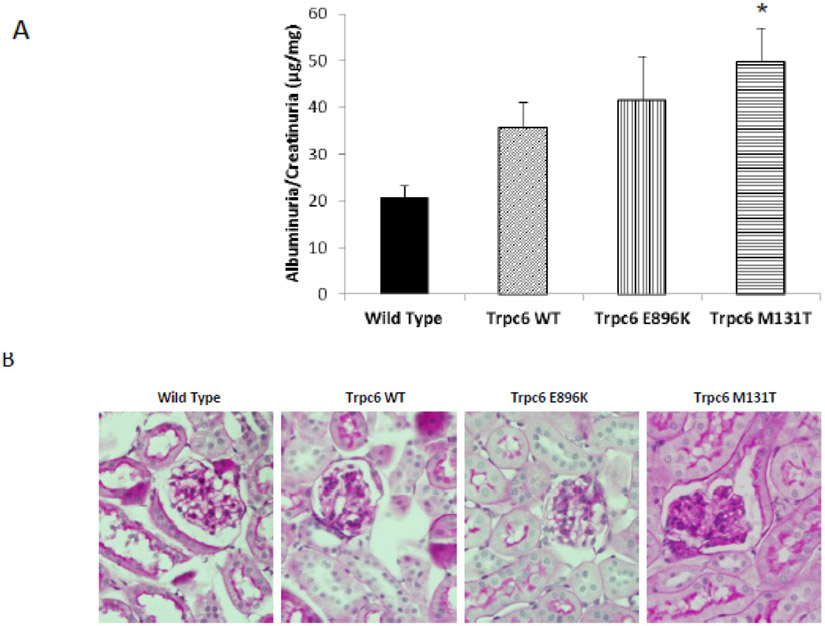
Early phenotypic characterization of three independent Trpc6 transgenic lines (A)Albuminuria (μg/dL) normalized by creatininuria levels (mg/dL) were tested in male mice at 2 month of age (wild type n=15, transgenic Trpc6 Wt n= 19, Trpc6 E896K n=9, Trpc6 M131T n=18). Data are presented as mean +/− SEM; ** p<0.01) (B) PAS staining of representative histopathological lesions in transgenic mice at 2 months of age. (400x). Sections were analyzed blinded to the genotype by a pathologist

Increased albuminuria/creatininuria ratio (proteinuria) is usually the first evidence of podocyte dysfunction. Since the pediatric patient carrying the M132T mutated form of TRPC6 developed an early onset of FSGS, we analyzed proteinuria in wild-type and transgenic mice at two months of age. We found a significant increase in albuminuria/creatininuria values in all the analyzed transgenic lines at two months of age when compared to the wild type mice, being Trpc6 M131T, the line that showed a biggest difference for this parameter. This fact provides insights of an early increased susceptibility to develop proteinuric disease in these transgenic mice (Fig. 3A).

In humans, TRPC6 mutations have incomplete penetrance and thus do not cause FSGS in all the individuals carrying the mutation. To assess the penetrance of proteinuria in our mouse models each individual mouse was considered proteinuric when the urine albumin/creatinine levels were higher than the average value of wild-type littermates plus 2 standard deviations. We observed that penetrance of this phenotype is stronger in the Trpc6 M131T mutant mice (Table 1).

To determine whether the increased albuminuria in transgenic mice was associated with enhanced renal injury, kidneys of two months old mice were systematically examined for the presence of pathological changes in the glomerular, vascular, and interstitial compartments. We scored in presence and severity the most common histopathology parameters found altered in glomerular disease, such as glomerular hypercellularity (HC), thickened membranes (MES) and focal and segmental glomerulosclerosis (FSGS). Glomerular lesions in the wild type group were relatively mild (Table 1). Kidneys from the transgenic mice presented an increment of pathological changes, but for most of the cases, those increases did not reach a significant difference when compared with their wild-type littermates. In contrast, Trpc6 M131T transgenic mice showed more severe pathological findings in glomeruli with significant increases in FSGS features (Fig. 3B, Table 1). Also, as shown in (Table 1), the global severity of renal pathological abnormalities (total score) is increased in Trpc6 M131T transgenic mice.

### 3.3 Wnt Signaling Pathways Appear to be Overactive in Mice over Expressing Trpc6 M131T Mutation

**Fig. 4.**
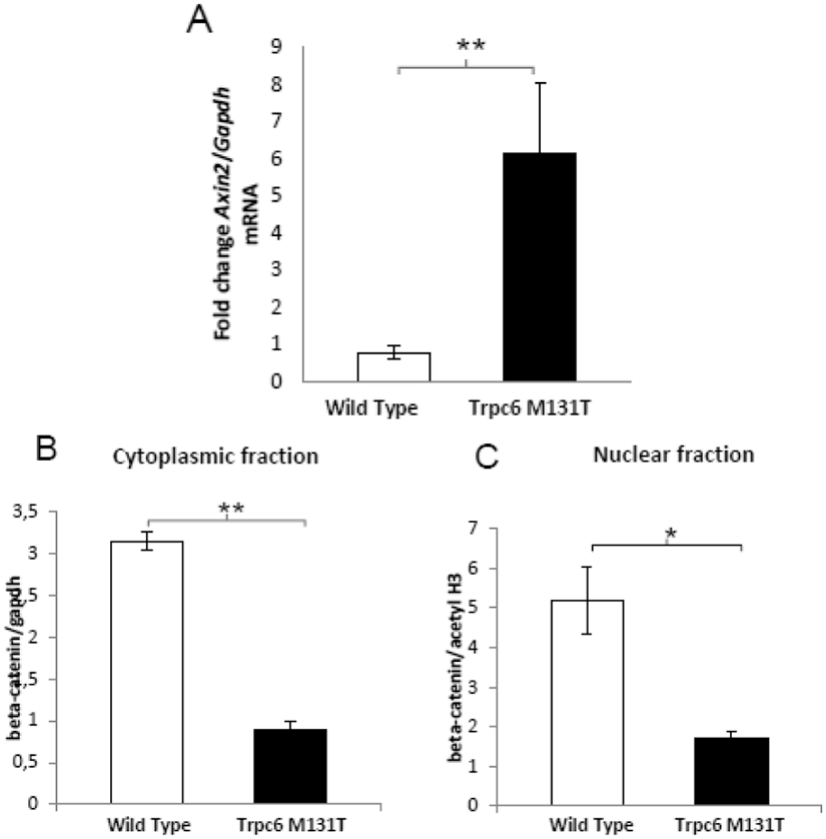
Molecular consequences of Trpc6 M131T over expression (A) Axin2 mRNA expression levels in Trpc6 M131t mice compared to wild type animals. Total RNA was isolated from an enriched glomeruli fraction. Real time PCR determined levels were normalized by Gapdh mRNA. (B) β-catenin nuclear translocation Western blot analysis. Cytoplasmic and nuclear protein fractions form enriched glomeruli were run in a 12% SDS-PAGE gel. Gapdh and acetyl-H3 were utilized as loading control for cytoplasmic and nuclear fractions respectively. The bands were digitally quantified and a ratio analysis between both fractions is shown. Data are presented as mean +/− SEM. * p<0.05; n=5

Given the convenience of analyzing a mouse model that manifests with an early onset plus a stronger phenotype of FSGS, we further explored the molecular pathways related to Trpc6 overexpression in glomeruli. We studied known canonical pathways important for normal kidney function, such as the Wnt signaling pathway. Calcineurin-NFAT and Wnt pathways have been largely described as key players relevant to normal kidney function in mouse models. For instance, the conditional activation of one NFAT isoform caused a kidney phenotype resembling FSGS through the upregulation of an NFAT signaling and the molecular alteration of the Wnt signaling pathway [28]. In order to determine whether the Wnt signaling pathway was altered in Trpc6 M131T mice, we analyzed the expression levels of *Axin2*, a known regulator of Wnt signaling, which when activated, reduces the β-catenin stability and consequently cell-cell adhesion. As can be seen in (Fig. 4A )*Axin2* mRNA levels were significantly upregulated in the glomeruli fraction obtained from the mutant mice compared to the wild type littermates (*p*<0.05). In addition, by Western blot analysis we identified that the β-catenin levels were significantly decreased in the Trpc6 M131T transgenic mice, both in the cytoplasmic and the nuclear fraction, (Fig. 4B-C) (*p*<0.05), suggesting that this pathway may be altered in Trpc6 M131T transgenic mice.

### 3.4 Most of the Described TRPC6 Mutations Associated with FSGS Increase the Channel Activity

**Fig. 5.**
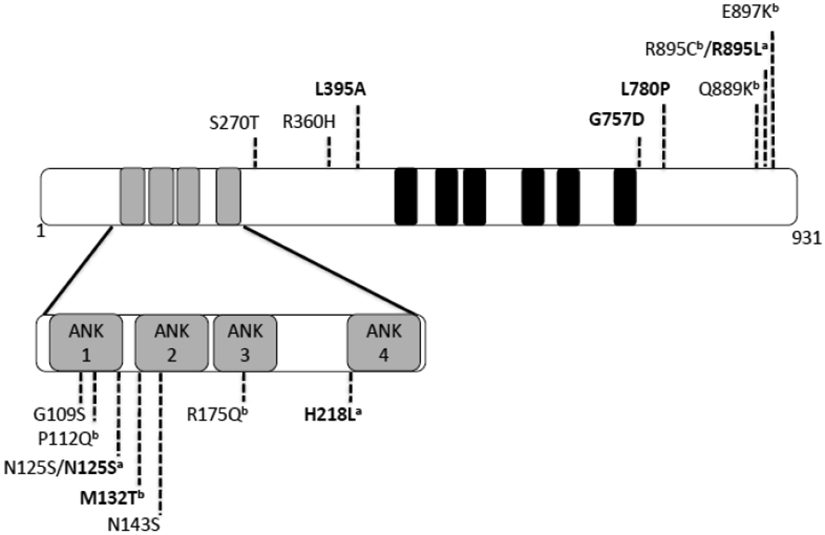
Schematic representation of the 931 aa long TRPC6 protein structure and point missense mutations associated with FSGS The structure of the human TRPC6 protein is depicted. Black boxes represent the transmembrane domains while gray boxes represent ankyrin domains. Mutations in bold have been found associated with FSGS of early onset while all the mutations showed in normal fonts have been found associated with late onset FSGS. For missense mutations tested in in vitro experiments, labelling is as follows: ^a^ presented increased levels of intracellular calcium; ^b^ showed an increase in current amplitude

Taken as a whole, all the analyzed Trpc6 transgenic mice lines appear to replicate the human mutated TRPC6 phenotype; increased proteinuria and histological FSGS features, which seem to correlate with the age of onset in human patients. Moreover, the incomplete penetrance which is defined as the percentage of individuals with a given genotype who exhibit the phenotype associated with that genotype, and that has been described in patients carrying Trpc6 mutations, seems to be also present in the analyzed Trpc6 transgenic lines.To date, a total of 17 TRPC6 missense mutations have been described in familial and sporadic FSGS cases [5,13,15-17,29-33]. Interestingly, seven out of these 17 mutations have been associated with a glomerular disease of early onset and 3 of them are localized within an ankyrin repeat in the amino-terminal region of the TRPC6 protein. However, after a domain mapping analysis for every single mutation, we did not find any correlation between the severity of the disease (early vs. late onset) and the localization of the missense mutation, neither considering that the mutation had been found inside or outside of a domain nor that the mutation was localized at the N-terminal or C-terminal region. Even more, the TRPC6 mutation N125S, has been detected in a case of early onset as well as in a case of late onset, making it difficult to predict the severity of the disease or the age of onset based on the position of the mutation (Fig. 5). However, in most of the cases the TRPC6 mutations associated with FSGS seem to increase the channel activity in one way or another. *In vitro* experiments have been performed for 11 of the 17 missense TRPC6 mutations in order to assay their effect on ion channel function. Studies have revealed that six mutations caused an increase in the current amplitude and three mutations caused an increase in the intracellular calcium (Fig. 5). This is very important and makes the increased activity of the channel the main target for any future drug development.

## 4. DISCUSSION

The existing knowledge on FSGS and understanding of glomerular function have been achieved mainly thanks to the development of different animal models during the last 15 years. Although these models have provided important highlights into glomerular diseases, they present some disadvantages when studying genetic forms of FSGS. For instance, some models exemplify acute processes that trigger FSGS (5/6 nephrectomy), whereas the damage in human patients is presented in a slow progression manner. In other circumstances, they illustrate different pathways that trigger FSGS because of a primary defect such as tubulointerstitial damage (adriamycin and puromycin-induced FSGS) or secondary forms of FSGS (HIV-1 infection). While these models contribute to the general understanding of end stage glomerular diseases, they do not confer new knowledge to the understanding of idiopathic/primary FSGS. Hence there is an urgent necessity of a mouse model that recapitulates chronic FSGS to efficiently study idiopathic/primary (reviewed by de Mik et al.) [34].

From this perspective, transgenic mice, although limited to one gene, are valuable tools to understand genetic forms of FSGS. With this goal, we have previously reported the generation of mouse models overexpressing either the wild-type or mutated forms (E896K and P111Q) of Trpc6 in mouse podocytes. We have shown that podocyte dysfunction is the principal cause of familial FSGS related with these two TRPC6 mutations. Interestingly, our results demonstrated that elevated levels of wild-type Trpc6 protein are sufficient to trigger proteinuria, histological and ultrastructural changes consistent with a FSGS phenotype and podocyte depletion [21]. However, these over expressing point mutations models (E896K and P111Q), as well as the overexpression of wild-type Trpc6, showed the disadvantage of presenting a kidney disease of late onset (5-9 months) making them an expensive tool for investigating the mechanisms involved in the pathogenesis of FSGS due to mutations in Trpc6.

In this report, we describe the generation and phenotypic characterization of an early onset FSGS mouse model due to M132T point mutation in Trpc6 calcium channel. Since the mouse Trpc6 protein is 1 aminoacid shorter than the human orthologous, the aminoacidic change M132T described in the human condition [15] corresponds to M131T in the mouse Trpc6. However, as described before, both orthologous proteins show a high homology percentage of 96% [21]. Trpc6 M131T transgenic mice present a kidney phenotype with an early onset and robust development of FSGS features. In addition, we provide *in vivo* evidence that the overexpression of this point mutation might cause NFAT and Wnt signaling pathways alteration. The Wnt signaling, Axin 2 mediated, was significantly upregulated in glomeruli form transgenic Trpc6-M131T mice. This fact is consistent with previous reports where the activation of the NFAT signaling in podocytes causes glomerulosclerosis through the Wnt signaling, which was also found altered as result of NFAT activation [28]. However, whether the Wnt / β-catenin pathway is directly involved in the pathogenesis of FSGS phenotype caused by Trpc6 mutations is a completely unknown issue in the field and its direct involvement remains to be elucidated. Nevertheless, based on this idea, we believe that the inhibition of NFAT signaling in podocytes might play an important role in the treatment of FSGS. The use of calcineurin inhibitors as cyclosporine A to block the NFAT activation and achieve an antiproteinuric effect [25], deserves to be explored in this transgenic mouse.

From this perspective, it will be interesting to test the therapeutic effects of known drugs such as FK506. TRPC6 seems to form heteromultimer complexes with TRPC3 and TRPC7, and the administration of FK506 in this transgenic mice model emerges as a good option, since it has shown to decrease proteinuria in a pharmacologically induced model of nephropathy by triggering the down regulation of TRPC6 and calcineurin, but also performs acute regulation of TRPC channels [35].

## 5. CONCLUSION

In summary, the presented model becomes a new tool to further explore the molecular basis and pathways involved in mutated Trpc6 based forms of FSGS and to create new drug discovery testing for idiopathic/primary FSGS.

## Figures and Tables

**Table 1.** Histopathology injury scores from kidneys of Trpc6 lines at 2 months of age

Line	Proteinuria levels (ug/mg)	Proteinuriapenetrance		Histopathology		
			HC	MES	FSGS	Total score
Wild type	20.7 +/− 2.5	-	4.4 +/− 0.6	2.8+/−0.5	0.8 +/− 0.4	8.0 +/− 1.2
Trpc6 Wt	35.6 +/− 5.5	21%	4.7 +/−0.5	2.5 +/−0.5	1.2 +/− 0.6	8.4 +/− 1.3
Trpc6 E896K	41.5 +/− 9.3	44%	4.8 +/−0.8	2.8 +/−0.7	1.4 +/− 0.7	9.0 +/− 2.0
Trpc6 M131T	49.8 +/− 6.9 (*)	55%	5.7 +/−0.4	2.7 +/− 0.4	2.5 +/− 0.5 (**)	11.9 +/− 1.1

Data are presented as mean +/− SEM; (Wild Type: n=9; Trpc6 WT: n=11; Trpc6 E896K: n=5; Trpc6 M131T: n=11).

** p<0.01
